# Consumption Curable; and the Manner in Which Nature as Well as Art Operates, &c.

**Published:** 1834-07-01

**Authors:** 


CONSUMPTION CURABLE; AND THE MANNER IN WHICH NATURE AS WELL AS REMEDIAL ART OPERATES, &c. &c. By *Francis H. Ramadge*, M.D. F.L.S. Octavo, pp. 168. Four Plates. March, 1834.

To those who are practically acquainted with the subject of this volume, the first line of the title-page—"CONSUMPTION CURABLE"—will convey an impression not very favourable to the work; but when it is remembered that its author was the public defender of St. John Long and his quackeries—the advocate of an illiterate Charlatan, who had been tried for manslaughter, or rather for womanslaughter—scepticism, if not something worse, will assume the ascendant. But although Dr. Ramadge has few claims on the forbearance of either ourselves or the profession at large, we shall endeavour to dismiss from our minds all circumstances and reminiscences respecting the author, and examine the work solely on its own merits.

Our author finds fault with the late Dr. Young for giving a discouraging opinion respecting the curability of consumption. Dr. Y. thought that not more than one case in a thousand recovered without assistance—and probably not more than one in a hundred, even with the aid of medicine. The word consumption includes so many grades and kinds of disease, that little satisfactory can be gleaned from any numerical calculation on this subject. If by consumption, we mean tubercular excavation or ulceration, then we aver that Dr. Young's calculation is, by no means, too unfavourable. And if various other morbid states and conditions of the lungs are included, then there is a chaos of uncertainty, of which Dr. Ramadge appears to avail himself in support of his "CONSUMPTION CURABLE." But what shall we say to the following statement?

"It is my intention, in the succeeding pages, briefly to shew that this statement is unfounded in fact; and that medical treatment has, in not a few instances, tended to prevent, rather than advance recovery. I trust I shall demonstrate, in the histories of some of my cases, that recovery has been owing, in a great measure, to the supervention of some catarrhal disease occurring spontaneously through an apparently imprudent exposure to cold, after remedial agents had failed." *Introd.* iii.

First it is insinuated that medical treatment not unfrequently prevents recovery, and then we are told that catarrhal inflammation supervening on consumption is a remedy for the disease!!! This brilliant idea—this real *novelty*—is followed up, and appears to be the vital principle of the whole work!! Speaking of Dr. Young, the author remarks:—

"Had this hospital physician been accustomed to the examination of dead bodies, he would have discovered, in more than one-fourth of the adult subjects examined after death, cicatrizations indicative of cured consumption; and on finding these appearances, had he enquired of some near relative of the deceased if the individuals, at any period of their lives, had for a time expectorated blood—had been troubled with indomitable cough, night sweats, diarrhœa, with emaciation of the body—or had been regarded by their medical attendants as consumptive, he would probably have arrived at a more favourable conclusion." iv.

So, then, *one-fourth* of the adults who die of other diseases have been previously cured of consumption!! In another place Dr. R. tells us that one-fourth of those who die, die of this disease: thus, as a fourth *die* of consumption, and a fourth are *cured spontaneously* of phthisis—leaving out of the question the great numbers saved by St. John Long and Dr. Ramadge, it follows that more than half of our population have consumption some time or other in the course of their lives!!

In respect to these "cicatrizations" indicative of "cured consumption," we may observe that some pathologists who are very anxious to find in the dead body what they are searching for—or what they predicted beforehand, will sometimes discover little puckered portions of lung which, to St. John Long—or to a writer on the sanability of phthisis, are a real God-send;—but have no more to do with consumption than with elephantiasis.

Our sapient author wonders that medical men have not noticed the non-liability of asthmatic people to consumption—and "that various species of catarrh are the instruments by which Nature chiefly arrests the disease, (phthisis.)"

"From long observation of the asthmatic state of the lungs, which is frequently witnessed on the dissection of persons, who, having recovered from consumption, have died from some other cause, I am induced to recommend artificial means to be early employed for the healing of ulcerous phthisis. After the careful examination of at least three thousand dead bodies, and after having had under my care many thousand consumptive cases, my fixed opinion is, that ulcers of the lungs are most effectually cured, and a fresh formation of tubercles prevented, by an expansion of the vesicular structure of the lungs; which it will be seen hereafter is, in many instances, brought on by chronic catarrh." vi.

This theory of catarrhal expansion of the air-cells of the lungs is a most happy and ingenious one for the practice in Harley Street—the artificial expansion by St. John Long's secret inhalations! The whole volume, indeed, shews that this most fortunate discovery was made, as it were, providentially, for the elucidation of the astonishing success of the ignorant quack and the learned FELLOW!

A theory, to be complete, must be tested in various ways. Thus the pathology must be corroborated by the etiology—and both, by therapeutics. Expansion of the air-cells (whether by accidental catarrh or St. John Long's inhalations) cures or prevents "CONSUMPTION." What occasions consumption? Why, contraction of the air-cells, of course—and this contraction results from the variable atmosphere of England—and the want of strong inhalations. Hence the salubrity of Harley Street—and the efficacy of the inhalers there!

"Now, when we take into consideration the peculiarly delicate conformation of the lungs, and their immediate susceptibility of every alteration in the atmosphere, we at once arrive at a resolution of the question. It is essential in order to maintain a healthy action and proper configuration of the chest, that our inspiration should be uniformly deep and full; but from the great inequality of atmospheric pressure resulting from the constant fluctuations of the weather, the depth and fulness of the inspiration are exposed to frequent diminution; and that play of the chest which is as requisite to a healthy state of the lungs, as exercise to muscular development, is consequently subject to repeated checks. Thus owing to the want of due excitement, or more strictly speaking of proper exercise, the healthy functions of the chest become deranged, its expansion is restricted, its action languid, and by degrees its shape alters, so that instead of the bony compages of the chest being forced boldly out in a somewhat semi-circular form, and the sternum pushed forward, the ribs fall in, drawing the breast-bone backward in a situation nearer the spinal column than is the case in its natural movement. Now to bear out and verify the foregoing remarks by shewing how requisite the expansible power of the chest is to the healthy constitution of the lungs, I would state as almost an invariable law that the commencement of pulmonary consumption will be found to take place in the superior lobes of the lungs, owing doubtless to the small extension of the upper ribs as compared with the more complete movement of the lower. Another singular instance confirmatory of the *novel view I am now taking of the subject,* is to be found in the exemption of asthmatic subjects from consumptive disorder. From the peculiar nature of their complaint, gasping for breath, and forced to *inhale* frequently, their lungs are ever fully exercised, and the expansion of the chest which follows as a necessary consequence, preserves the sufferer free from the attacks of this still more dreadful malady." 7.

In justice to the author we must say, that *we* have marked in Italics what his own modesty would not permit him to lay an emphasis on—the *novelty* of the doctrine, and the utility of *inhalation.*

Dr. R. extends this doctrine to various classes of society—to the care-worn—to the victims of disappointed love, blighted ambition—and to those, "whose distemper *improper medical treatment* has unduly prolonged or confirmed." Shade of Miss Cashin! didst thou not, if permitted to wander in these upper regions, cross the mental horizon of Dr. Ramadge, the prompter and advocate of St. John Long, when he penned this last sentence? It seems that thou didst not—for the insinuation respecting *improper* medical treatment is not levelled against the *excoriator* in Harley Street, but against Dr. Ramadge's own professional brethren!

And how are the above-mentioned victims made to illustrate the new theory? Very easily. They are unable "to take inspirations of depth sufficient to keep up the necessary changes produced by the air on the venous circulation." Hence the necessity for expanding the lungs by St. John Long's inhalers.

"Following up the tenor of the preceding observations, we shall find that the benefit usually derived from a sea voyage, or change of air, is not so much due to the removal from an impure to a purer atmosphere *per se,* but to the stimulating effects produced on the respiratory organs, and the increased energy of the muscular powers of the chest, on which pulmonary dilatation is of course consequent." 9.

According to this doctrine, the consumptive patient should be sent to the Alps or the Highlands, there to inhale pure and stimulating air, while expansion of the air-cells would result from climbing the mountains!

We may pass over two short chapters on the common causes and the ordinary symptoms of phthisis; but the following highly valuable and most easy diagnosis of "CONSUMPTION" must be recorded in our pages for the good of humanity.

"The plan I always pursue, and indeed a most ready one, to distinguish consumption from pulmonary catarrh, with which it is most liable to be confounded, is to apply the ear to the posterior part of the chest, about two or three inches below the inferior angle of the scapula. Here, if the respiration be almost natural or slightly puerile, we can at once, and early, proclaim the case to be phthisical, though the patient may have a troublesome cough, and but few of the common symptoms belonging to consumption." 24.

Now, if natural, or almost natural respiration, two or three inches below the scapula, be an unfailing mark of phthisis, we do not wonder at the "sanability of consumption," both in Harley Street and Ely Place!

Passing over the chapter on morbid appearances, where we could not expect much new matter after the labours of Laennec, Bayle, and our own pathologists, we come to the fifth chapter, on prophylaxis.

Even before St. John Long made his appearance in the medical horizon, it appears to have been the practice of our author to prescribe generous living and invigorating diet to those who had any tendency to consumption. A sea voyage was also advised, with the hope that the saline particles in the marine atmosphere might act as a "stimulant to the lungs," and thus render the inspirations deeper. Another great hope from a sea voyage is the chance of the patient's *catching cold*—which is quite a god-send with Dr. Ramadge, inasmuch as the establishment of a "slight permanent catarrh" enables the patient to "bid defiance to phthisis." A stumbling block to the new doctrine is found in the fact that those musicians who play on wind instruments are peculiarly subject to consumption. But our ingenious author gets rid of this by the following reasoning.

"Whoever will take the trouble to watch attentively a player on the flute, or clarionet, &c. will find that although the performer seemingly inspires and expires frequently and fully, yet that in point of fact he often makes several consecutive expirations to one inspiration. Thus his breathing, so far from being advantageous, and so far from developing the lungs, as I have previously declared well-regulated mechanical exercise will ever do, is indeed so irregular, and furtive, that it produces effects entirely the reverse, narrowing the chest, and confining the volume of the lungs." 71.

Well done. The flute-player injures his lungs by blowing out a great deal more air than he draws in! No wonder that he *expires* in the end by consumption!

We were a little amused at finding Lord Eldon among the *causes* of consumption, and Lord Brougham on the list of prophylactics. The reader will long for an explanation. Here it is.

"We are accustomed to look back with horror on the proceedings, recorded by historians, as having taken place in that iniquitous court, which went under the name of the Star Chamber. How then, judging by analogy, will our posterity execrate the records left them of the practice of the Court of Chancery. They will there read a piteous tale of justice withheld, of hope, the brightest boon of heaven, extended and protracted, until to look forward is to exclaim with Lear, "oh! that way madness lies." The ghastly train of diseases, consumption, cancer, and other fell destroyers of the human race, which I have seen brought on the wretched victims of the procrastination, until recently, the characteristic of this court, leads me in common with the general voice to reflect with gratitude on the change that has been effected by the wise energy of one master-spirit. Had the reforms introduced by him, been adopted even a few years sooner, how many a fair fabric of human happiness would have been spared, that now lies dismantled, and overthrown!" 88.

After the specimens which we have given of our author's *doctrines*, we apprehend that our readers will not wish for a very extended account of Dr. Ramadge's *practice* in consumption. "There are but two modes by which we can hope to cure this disease: the one by rendering it chronic, and the other *by artificially enlarging those portions of the lungs which are pervious to air.*" It is our author's great aim "to put an end, as early as possible, to the symptoms of hectic fever." This he successfully accomplishes, in many instances, by general and local bleeding. INHALATION is the other grand remedy. The principle on which this measure proves beneficial, is, our author avers, entirely misunderstood. It is not by the application of vapour or medicinal substances to the diseased lungs that good is done, but by the mechanical dilatation of the air-cells, and the expansion of the chest.

We shall make one more extract and have done.

"Should there be catarrh in the superior bronchial tubes, of a duration sufficiently long only to heal ulcerations and cure the patient, he may months or years afterwards, if there be any cause assisting to impair the general health, be again attacked by consumption; but never can this relapse happen if the bronchial tubes be subacutely inflamed for a period sufficient to produce chronic dyspnœa or habitual asthma more or less severe. Half of those which are commonly regarded as cases of asthma, originate in consumptive disease whose progress has been arrested by the supervention of that affection; but in which neither fresh crops of tubercles nor hectic fever need be apprehended. Any individual indeed having asthma, from whatever cause resulting, is as perfectly exempt from consumption, as he who had been consumptive, but has afterwards had his disease merged into asthma. In a word, it may be confidently affirmed, that no asthmatic person need ever be afraid of becoming consumptive.

In order to promote expansion of the aërial tissue of the lungs, it is my usual practice in the absence of catarrh, and when congestion in the chest, and the symptoms of hectic fever have been diminished by small general bleedings, repeated at proper intervals, or by the application of leeches over the second and third ribs anteriorly, to advise inhalation as soon as possible. A drawing of a suitable apparatus will be found at the end of this work. There are few early cases of consumption, but what will be rapidly improved by this treatment steadily pursued. The disease being thus checked, the same changes will follow which are attendant on catarrh. The nodules of unripe tubercles will become innoxious in consequence of being surrounded by black secretion, or what has been called black pulmonary matter; and small cavities already formed will have their surfaces soon brought in contact so as to heal by what surgeons term the first intention. It is, we must own, preferable to effect pulmonary expansion by sure artificial means, rather than to depend upon the uncertain production of catarrh. And there is another important point gained, inasmuch as recovery takes place unaccompanied by any cough or difficulty in breathing, which generally attends those cures which nature herself now and then accomplishes by introducing this less fatal, yet distressing complaint. Inhaling performed two or three times daily, for half an hour each time, will in the space of a few weeks work a wonderful change on the chest; externally the muscles concerned in respiration will be manifestly enlarged, and the bony compages of the chest both before and laterally visibly increased; whilst, at the same time, the natural respiratory murmur will be heard internally, far more distinct than ever. Such has been the increase of size which the chest, in young persons especially, has undergone through the exercise of inhalation, that I have known a waistcoat which buttoned easily require in little more than a month to be widened. It is in fact incredible to one who has never been at the pains to measure the chest, or examine its shape, what an enlargement it acquires, by the simple action of breathing for the time above stated, backwards and forwards, through a narrow tube of a few feet in length." 105.

Of the cases in this volume we shall take no notice. Mr. A——, Mrs. B——, Master C——, and Miss D——, are all very well for the non-professional reader—and for him or her alone are they designed. They will answer the author's objects—and that is enough. We forgot to mention our author's ideas as to *climate* in consumption. He prefers St. Petersburgh to Italy, "a thousand times"—because, "in the latter, he (the patient) has a chance of contracting catarrh, and of thus staying consumption." 132.

Of the plates we need say little. The first forms as good a representation of Vesuvius during an eruption, as any we have lately seen. A volume of flame is rising from the crater of the mountain, while torrents of molten lava are gushing from the side. Along the base are scattered fragments of pumice-stone, black scoriæ, and blue clay, such as we see in the neighbourhood of Herculaneum and Pompeii.

In this publication Dr. Ramadge has had sagacity enough not to mention the name of St. John Long, or allude to his cures. But he advocates the principles and practice of the quack—the stretching of the lungs by inhalation, and the restoration of the consumptive constitution by wine and good cheer. The Fellow of the Royal College of Physicians has set his professional brethren at defiance, and cast himself on the ignorance and credulity of the public. The reward at which the author aims is best known to himself; but we venture to predict that it will be any thing except professional reputation, in the true sense of the word.

